# Detail microscopic analysis of deep fascia of lower limb and its surgical implication

**DOI:** 10.4103/0970-0358.73424

**Published:** 2010

**Authors:** Visweswar Bhattacharya, Partha Sarathi Barooah, Tapas Chandra Nag, Gaurab Ranjan Chaudhuri, Siddhartha Bhattacharya

**Affiliations:** Department of Plastic Surgery, Institute of Medical Sciences, Banaras Hindu University, Varanasi, India; 1 Department of Anatomy, All India Institute of Medical Sciences, New Delhi, India

**Keywords:** Confocal microscopy, deep fascia, electron microscopy, flap, microscopic anatomy

## Abstract

**Background:**

The knowledge regarding the structural details of deep fascia remains inadequate. It was described to be relatively avascular having predominantly protective function. Anatomical and surgical studies revealed that it had associated vascular arcade and hence incorporated it to ascertain additional vascularity to the flaps. However, not much importance has been directed towards the detailed study of the various constituents of deep fascia in order to explain its physiological and clinical implications. Therefore, this study was undertaken to unveil these details.

**Materials and Methods:**

Fifty fresh specimens of human deep fascia overlying the gastrocnemius muscle were analyzed regarding the (i) vasculature, (ii) matrix, and (iii) other structural elements. The deep fascia was procured in three forms; (a) both the layers, (b) superficial layer, and (c) deep layer. Detail study was conducted by light, confocal, and electron microscopy.

**Results:**

Under light microscopy, blood vessels including capillaries were seen associated with both the layers. Perforators traversing the intra-fascial plane could be visualized. Confocal microscope optical sections showed well-organized bright fluorescent collagen fibers and nuclei of various cells. Electron microscopic evaluation revealed many interesting constituents which are relatively unknown to the anatomist and clinicians. There were arterioles, capillaries, venules, lymphatics, nerves, mast cells, and myofibroblasts apart from collagen and elastic fibers.

**Conclusion:**

The detail structural analysis of deep fascia provided the clue to its rich vascularity and other structural constituents. They all contribute to enhance the vascularity and maintenance of the physiological functions of fasciocutaneous, adipofascial, and fascial flaps, frequently used for reconstructions. Thus, incorporation of deep fascia in the flaps during reconstruction is highly beneficial for ensuring optimal vascularity.

## INTRODUCTION

Deep fascia is present in our body in different tissue planes and in different forms with specific function. This study is concerned with the fascia overlying the muscles of the lower limb. The standard textbooks of anatomy describe the deep fascia as comprising of fibrous tissue whose main function is to protect the underlying muscles. There is inadequate information in the literature regarding the structural details of deep fascia. This study analyzed its various constituents histologically. The deep fascia was examined under low power (LP) and high power (HP) microscope, followed by confocal microscopic study and then electron microscopic evaluation. Such extensive analysis revealed several interesting findings, which were not known till date.

One may argue that what is the utility of knowing the structural details of deep fascia? The answer is to attain an evidence based knowledge of why its incorporation in a flap improves the latter’s vascularity, and thereby, chances of survival. It is desirable to know the contribution of each constituent of a composite tissue used for reconstruction.

When routinely the fasciocutaneous flap is dissected without magnification, few observations are encountered: (i) a stable deep fascia is identified, (ii) there is a distinct loose areolar subfascial plane of dissection, (iii) underlying healthy muscle is seen, (iv) while dissecting the flap several perforators of variable sizes along with their venae comitantes are encountered and severed, and (v) the presence of rich subfascial vascular network is observed. However, we do not realize that during flap elevation, nerves and lymphatics are also being disrupted, as their caliber is much less than the perforating vessels. It may be argued that one need not give too much importance to deep fascia as it may lack other functional elements of a highly vascularized tissue and it acts only as a barrier structure between adjacent tissues. We however believe that the deep fascia is a highly vascular structure having various functional elements to maintain its different physiological requirements. Hence we undertook this detailed study.

## MATERIALS AND METHODS

The material of this study was fresh human deep fascia over the gastrocnemius muscle as it is very well delineated. Fifty specimens from 50 patients measuring 1 cm × 1 cm were procured from the adjacent area while harvesting a fasciocutaneous flap or during excision of a benign suprafascial lesion. The deep fascia over gastrocnemius was taken as a representative tissue although the thickness of fascia varies over different muscles. Forty of them were male and ten were female, with the age ranging from 25 to 40 years. The study was planned with three aims: (a) to examine the vasculature, (b) to investigate the details of matrix consisting mainly of collagen fibers, and (c) to find out the presence of other structural elements. These features were studied by the following parameters: (i) LP and HP light microscopic histological analysis, (ii) confocal microscopic study of fluorescenized deep fascia, and (iii) electron microscopic study.

The study was approved by the institutional review committee, and the subjects gave written informed consent.

### Histological study

Fifty specimens of deep fascia were procured. The specimens were outstretched on glass slides with the help of string and were fixed in 10% formaldehyde solution for 24 h. The fixed tissues were processed in automated tissue processor. The paraffin blocks were prepared. The sections were cut and stained using hematoxylin and eosin stain.

### Confocal microscopic study

Inferiorly based fasciocutaneous flap was dissected from gastrocnemius region in 30 patients. Then, adjoining proximal subcutaneous tissue was undermined to visualize the deep fascia. Then, three types of fascial flaps were dissected: (A) facial flap of 2 cm × 2 cm containing both the layers of the deep fascia. In the immediate adjacent area, two flaps of similar dimension were meticulously dissected with a common base (B) consisting of superficial layer of deep fascia, and (C) the deeper layer of deep fascia. The fluorescein sodium dye was injected systemically through a peripheral vein in the dose of 30 mg/kg body weight. Prior to injection, the sensitivity to the dye was tested. Twenty minutes after injection, these flaps were observed under the ultraviolet lamp making the room dark. Bright fluorescence was observed in each flap proving the presence of dye in the tissue through circulation. Then, these three flaps were detached and put in 4 °C stored 1× PBS (phosphate buffer solution) (F.N. 1) for 2 min. They were fixed with 7% paraformaldehyde (PFA) for 20 min and washed in 1× PBS. The specimens were immediately transported to the laboratory in the Department of Zoology. The tissue was gently spread over a glass slide and washed twice with 1× PBS for 10 min each. It was then mounted with an antifade solution (Molecular Probe, USA) for preventing quenching. The tissue was ready for optical section in confocal microscope.

### Footnote

Composition and preparation of 1× PBS (Phosphate buffer solution)

4 g—NaCl, 0.1 g—KCl, 0.72 g—Na_2_HPO_4_, 0.12 g—KH_2_PO_4_

- These components are dissolved in 400 mL of water. pH of the solution is adjusted to 7.4 by adding HCl and a total of 500 mL of solution is prepared.
- This solution is then autoclaved and is ready for use. 7% of PFA prepared by adding 7 g of PFA in 100 mL of 1 × PBS. This solution is heated on water broth to dissolve. Then, it is aligited and stored at 4 °C for 1 h for future use.

### Electron microscopic study

Fresh deep fascia was harvested in 20 patients consisting of (A) both the layers, (B) superficial layer, and (C) deep layer. The samples were fixed in a mixture of 2.5% glutaraldehyde and 1% PFA in 0.1 M phosphate buffer (pH 7.4) for 5–7 h at 4 °C. They were washed and stored in buffer at 4° C. The materials were transported overnight to the electron microscopy unit at All India Institute of Medical Sciences, New Delhi. The samples were postfixed in 1% osmium tetroxide for 2 h at 4 °C, dehydrated, infiltrated, and embedded in araldite CY 212. Semithin sections (1 *μ*m thick) were cut, stained with aqueous toluidine blue, and observed under an optical microscope for general survey. Ultrathin sections (70–80 nm) were cut, contrasted with uranyl acetate and alkaline lead citrate. They were finally viewed under a Morgagni 268 D transmission electron microscope.

## RESULTS

**Figure 1:**
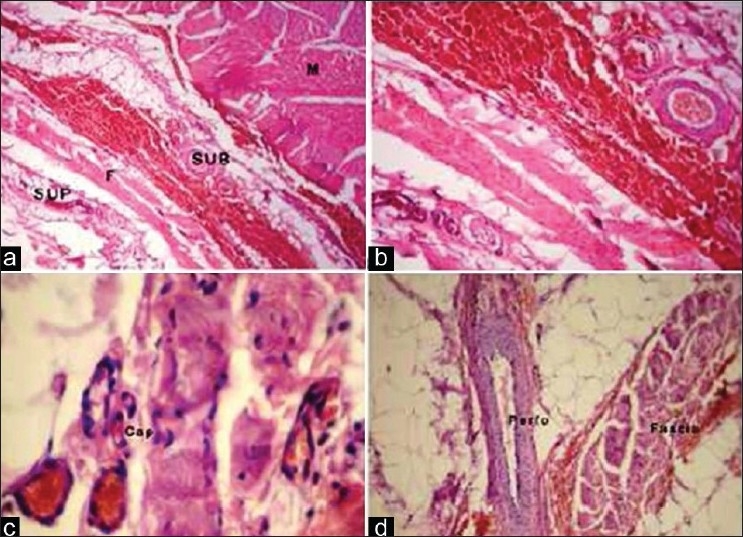
Photomicrograph of the deep fascia showing (a) Superficial and deep layer of fascia and skeletal muscle bundle (H&E, × 40). (b) Fascial vasculature (H&E, ×100). (c) Intrafascial capillaries (H&E, ×400). (d) The perforating vessel (artery) (H&E, ×100)

On light microscopic examination (10×), we could visualize the relatively loose superficial layer as compared to the tougher deep fascial layer. The deep layer had few elastic fibers. All types of blood vessels were observed in the superficial as well as deep layer of deep fascia [Figures 1a–c] namely, arterioles, capillaries, and venules. The perforators could also be observed in the intrafascial plane (under 40×), justifying their anatomical location as they pierce the deep fascia [Figure 1d].

**Figure 2:**
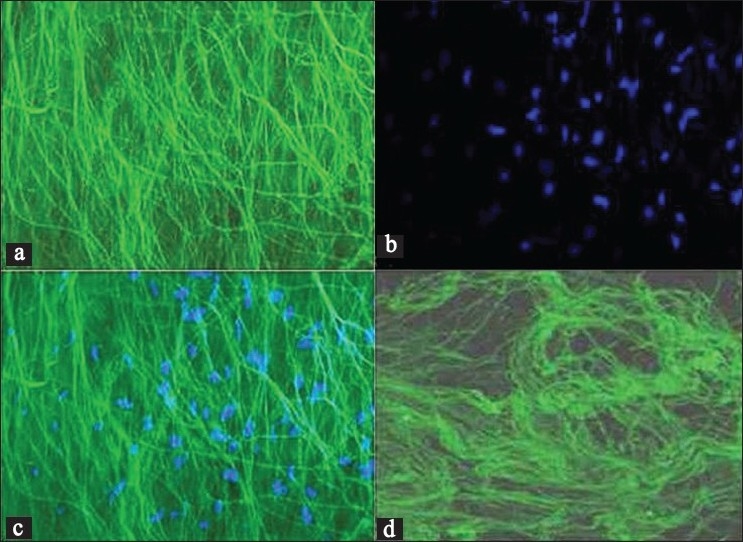
Confocal microphotograph of the deep fascia showing (a) collagen fibers on application of fluorescein dye. (b) Nuclei of the fibroblast. (c) Merged data of (a) and (b) giving the total picture of deep fascia. (d) Autofluorescence in collagen fibers

Deep fascia treated with fluorescein dye was scanned under a laser scanning multiphoton confocal microscope (Radiance 2000) using appropriate filter band (HQ515/30). Each optical section of the processed deep fascia sample was analyzed carefully, which showed bright fluorescent collagen fibers and fluorescent nuclei of the different cells [Figures 2a and b]. When all the optical sections were merged, it showed mixture of the matrix fibers and the nuclei of different cells entangled in it [Figure 2c]. The control sample without any fluorescein treatment showed autofluorescing collagen fibers but of lower intensity compared to the fluorescenized sample [Figure 2d].

On electron microscopy examination following interesting features were documented which are relatively unknown to majority of clinicians.

**Figure 3:**
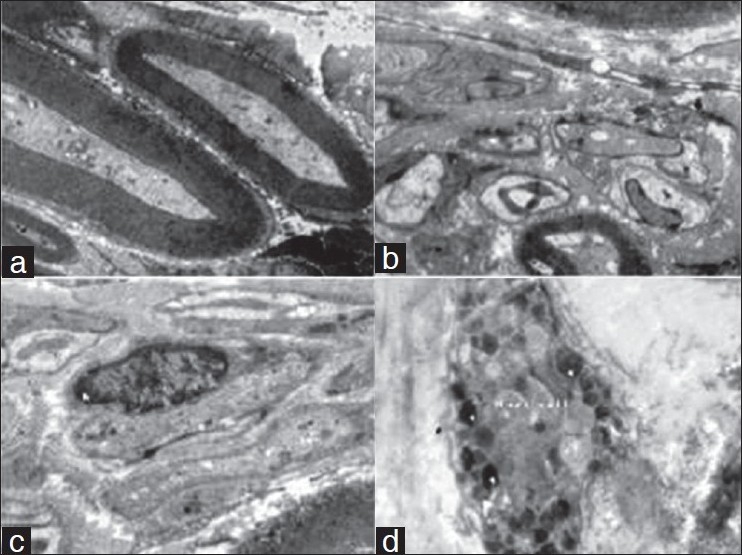
Electron microphotograph of the deep fascia showing (a) myelinated nerve axon (×4000). (b) Nonmyelinated nerve axon (×4000). (c) Schwann cell with nucleus (n) (×4000). (d) Mast cells with granules (g) and villous projection (f) (×4000).

**Figure 4:**
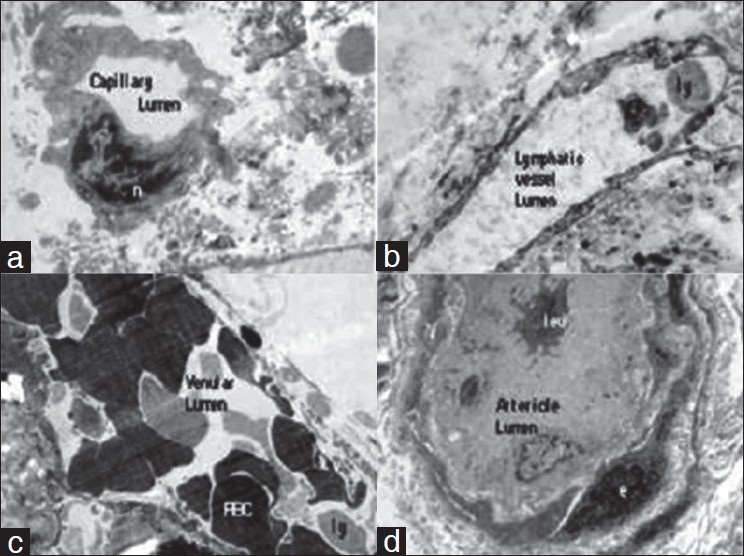
Electron microphotograph of the deep fascia showing (a) Capillary, the single endothelial cell nucleus (n) (×4000). (b) Lymphatic vessel with lymphocyte in the lumen (ly) (×4000). (c) Venule with lymphocyte (ly) and RBCs in the lumen (×4000). (d) Arteriole with leukocyte (leu) in the lumen (×4000).

**Figure 5:**
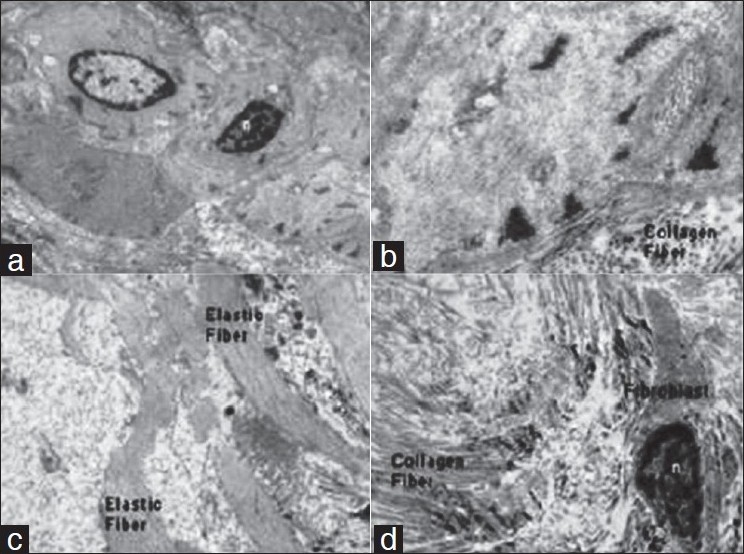
Electron microphotograph of the deep fascia showing (a) Myofibroblast and nucleus (n) (× 4000). (b) Myofiberfilament with transverse section of collagen fibers adjoining to it (×4000). (c) Elastic fibers (× 4000). (d) Collagen fibers and fibroblast showing nucleus (×4000)

- The nerve fibers (myelinated and unmyelinated nerve axons) and Schwann cells [Figure 3a–c].
- Mast cells [Figure 3d]
- Capillary channels [Figure 4a]
- Lymphatic vessels [Figure 4b]
- Venules [Figure 4c]
- Arterioles [Figure 4d]
- Myofibroblast and myofibrils [Figure 5a and b]
- Collagen and elastic fibers [Figure 5c and d]. The elastic fibers were more prevalent in the deeper layer.

## DISCUSSION

Standard text books of anatomy consider that the deep fascia consists of parallel bundles of collagen fibers, having a mechanical behavior similar to that of an aponeurosis.[1,2] In contradiction to common belief, several authors have demonstrated that deep fascia has rich vascular network. On the basis of this knowledge, it is preferably incorporated in the composite tissues for reconstruction.[3] This vascular arcade is predominantly supplied by the musculocutaneous and septocutaneous perforators.[4,5] While piercingthe deep fascia, they form subfascial vascular plexus. After coming out on the superficial surface, they form suprafascial vascular plexus. Subsequently, it communicates with the subcutaneous and subdermal plexuses.[6] Stecco *et al*.[7] described the intrafascial vascular network as well as the type and caliber of the vessels. They also mentioned that numerous vessels follow tortuous paths through the different collagen layers of the muscular fascia.[8] Thus, it maintains the continuity of the horizontal and vertical vascular network which enhances the vascularity and provides safety to the fasciocutaneous, adipofascial, and fascial composite tissue transfer.

We have intraoperatively demonstrated that the deep fascia of the lower limb consists of a superficial and a deep layer. Both these layers have independent-rich vascular network.[9] The deeper layer is having “subfascial vascular plexus” and the superficial layer contains the “suprafascial vascular plexus.” While dissecting a fasciocutaneous flap, one can easily visualize the subfascial plexus and while undermining the subcutaneous tissue the suprafascial plexus is witnessed. Combining both these layers in the flap adds undoubtedly to the latter’s vascularity.

While harvesting fasciocutaneous flap, deep fascia overlying the gastrocnemius muscle can be easily separated from both the underlying muscles as well as from the subcutaneous tissue. This study revealed that the deep fascia contains all tissue constituents necessary for maintaining the physiological functions. The dynamics of these structures are well established. The essence of this study is to explain how by incorporating the deep fascia in the flap, the vascularity, and other physiological functions are augmented. Stecco *et al*.[7] also mentioned that only recently the deep fascia has been studied from anatomical and histological point of view, and a correlation between the structure of the fascia and its mechanical features is required. The reconstructive surgeons are basically interested in adequate circulation, normal physiological function, and stable long-term behavior of the transferred tissue. The various elements of the deep fascia meet these goals.

The rich vascular arcade is comprised of arterioles, capillaries, and venules. Beyond arterioles, the capillaries are the eventual delivering medium for the nutrient/gaseous exchange in any tissue. The presence of capillary channels in the deep fascia establishes the vascular affluence and dynamic metabolic commotion of the deep fascia. Moreover, these capillaries were studded with RBCs signifying active blood flow through them. The intrafascial course of the perforator seen under light microscope confirms the continuity of these vessels as they communicate with the suprafascial plexus. In fact, they can be traced under loupe magnification as they pierce the subfascial surface. Such observations were also made by Schäfer[10] and Tolhurst.[11]

The deep fascia has high tensile strength and elasticity due to its parallel collagen bundles. In addition to rigidity, the fascia forms a gliding interface with the underlying epimysial capsule of muscle. This allows free excursion of the muscle under the relatively immobile skin and the fascia attached to it.[12] The hyaluronic acid present over the gliding surface makes this movement possible. Although there are no synoviocyte-like cells present on the deep fascia yet the deep fascia has capability to secrete hyaluronic acid.[13,14]

The intima of the lymphatics was lined by single layer of endothelial cell. Since we could demonstrate all type of vessels including the lymphatic channels [Figure 4b] in deep fascia, it provides good reason for fluid movement on the deep fascia surface. The information regarding presence of lymphatics in the deep fascia is scanty in the literature. The venules and lymphatics also drive out the fluid from the tissue maintaining proper circulation and reducing the oedema of the flap.

The collagen and elastin fibers of the deep fascia matrix vary. The deep layer has scanty elastin fibers. The confocal microscopic study of the matrix revealed collagen bundles and the nuclei of the different cells (myofibroblast/fibroblast/endothelial cells, etc). Most of the collagen fibers are oriented in one direction. Stecco *et al*.[8] also observed two to three layers of parallel collagen fiber bundles which are separated from each other by loose connective tissue. The tensile strength of the fascia is dependent on the matrix content. This allows strong resistance to pressure and traction. They are also responsible to adapt to volume variations of muscles during their contractions. As the age progresses, the tensile strength of the deep fascia reduces remarkably (degenerative changes), justifying the changes in the active physiological process.[15]

The demonstration of myofibroblast nuclei by electron microscope in deep fascia suggests its toughness and contractile ability. This contractile property should be modulated by an adequate neural supply. In our study, the demonstration of myelinated, nonmyelinated nerve fibers, and Schwann cells in the deep fascia by the electron microscopic examination corroborate this notion. Some authors have also mentioned that the nerve fibers are homogeneously distributed throughout the fibrous components of the fascia.[8] The presence of mast cells in any connective tissue is not an unusual phenomenon, but we rarely come across the report of their presence in the deep fascia. However, their presence affirms the protective role of deep fascia.

In conclusion, this study suggests that the deep fascia is not merely a strand of collagen and elastin fibers acting as barrier structure, but a metabolically very active, serving as a tough protector, providing gliding surface, elastic, contractile, sensitive, and highly vascular unit. Therefore, its inclusion in the flap is structurally and functionally highly beneficial.
